# Special Issue: Fungal Nanotechnology

**DOI:** 10.3390/jof7080583

**Published:** 2021-07-21

**Authors:** Kamel A. Abd-Elsalam

**Affiliations:** Plant Pathology Research Institute, Agricultural Research Centre, Giza 12619, Egypt; kamelabdelsalam@gmail.com; Tel.: +20-010-9104-9161

## 1. Introduction

Fungal nanotechnology (FN) or myconanotechnology is a novel word which was originally introduced in 2009 by Rai M. from India (Myco = Fungi, Nanotechnology = material production and utilization in the 1–100 nm size range). It is described as the manufacture and subsequent use of nanoparticles via fungus, especially in biomedical, environmental and agricultural commodities [1,2]. FN investigates numerous syntheses of metal nanoparticles: processing techniques, preservation of the environment and prospects of the future. Certain nanomaterials, such as silver, magnesium, gold, palladium, copper and zinc, have also been found, such as selenium, titanium dioxide, metal sulfides, cellulose, and other key fungal species, including mushrooms, Fusarium, Trichoderma, endophytic fungus and yeast. Studying the actual process of nanoparticle production and the impact of various variables on metal ion reduction might assist in developing cost-effective synthesis and nanoparticle extraction techniques. It will also address mycogenic nanoparticles, risk assessment, protection and control. Fungi have the ability to generate many extracellular enzymes that hydrolyze complicated macromolecules and to produce a hydrolyte in the wake of these enzymes. The metabolic capability of its usage in bioprocesses has been a strong source of concern for the application of fungus as a main producer for various types of metallic NPs [3,4].

For example, *Rhizopus oryaze* metabolites were utilized as a biocatalyst for the green synthetization of magnesium oxide (MgO-NPs) nanoparticles [5]. Additionally, biogenic selenium nanoparticles (Se-NPs) was produced by *Bacillus megaterium* and was used as an antifungal agent against *R. solani*, the causal organisms of damping and root rot disease in *Vicia faba*, as well as for induction of plant growth [6]. Bacillus-mediated AgNPs with an onion-isolated endophytic bacteria Bacillus endophyticus strain H3, bactriosynthesized AgNPs with a concentration of 40 μg/mL, had a high rice-blast antifungal activity with an inhibition rate of 88% mycelial. In addition, spore germination and *M. oryzae* appressorium have been considerably suppressed by AgNPs [7]. The Fusarium genus, one of the most common fungal species, plays an important role and can be considered a nanofactory for the production of various nanoparticles. Fusarium spp. is a type of fungus. This issue discusses the production of silver nanoparticles (AgNPs) from Fusarium, as well as its mechanism and uses [8]. Although the function of lichens as natural factories for synthesizing NPs has been documented, the production of NPs using lichens has remained completely unexplored until now. Lichens have the ability to produce several forms of NMs, such as metal and metal oxide NPs, bimetallic alloys and nanocomposites, through a reducible activity [9]. Mycofabrication can be described as the synthesis of various metal nanoparticles via fungal species. Several fungal species are eco-friendly, clean, non-toxic agents for the synthesis of metal nanoparticles, using both intracellular and extracellular methods.

Due to the vast spectrum and diversity of fungi, the mycogenic synthesis of nanoparticles, an essential element of myconanotechnology, leads to an intriguing new and practicable multidisciplinary science with significant results [10]. Myconanoparticles have been used extensively to detect and control pathogenic agents, to clean wastewater, to conserve food, as nematicides and for many other products. The mycogenic nanoparticles produced by various fungal species may be used in some potential agricultural applications to improve crop production by increasing growth and protection from infections. In addition, this will improve the toxicity to plant ecosystems of chemical pesticides, insecticides and herbicides [11]. In the near future, myconano-functional agrochemical products, crop protection pre-harvest and post-harvest, sensing systems and genetic equipment will be enhanced for direct use in farms and fields [12]. Fungal-mediated nanoparticles have shown effective inhibition against pathogens causing infectious diseases in humans, especially against those deemed multi-resistant to traditional antibacterial agents [13]. Fungi-based nanosorbents are also a novel study path in the field of heavy metal biosorption from wastewater pollutants [14].

Fungal-mediated nanoparticles have been used effectively in a wide range of scientific domains, including medicines, pharmaceuticals, agriculture, and electronics. As a result, some evaluations concentrated on the application of mycogenic nanoparticles against plant diseases, post-harvest antibiotics, mycotoxin management, and plant pests, as well as certain animal pathogens. Furthermore, fungal nanomaterials have a high potential and promise for enhanced diagnostics, biosensors, precision agriculture, and targeted smart delivery systems. For example, soil mycobiota can influence zinc mobilization from ZnO NPs in soils and thus zinc mobility and bioavailability. As a result, *Aspergillus niger,* a common soil fungus, was chosen as a test organism to evaluate fungal interactions with ZnO NPs. As expected, the *A. niger* strain had a significant effect on the stability of particulate forms of ZnO due to the acidification of its environment [15]. The macrofungi-derived NPs produced by major mushroom species such as *Agaricus bisporus, Pleurotus* spp., *Lentinus* spp. and *Ganoderma* spp. are widely recognized to have strong nutritional, immune-modulatory, antibacterial, antifungal, antiviral, antioxidant and anticancer activities [16]. In addition, the existing and potential applications of zinc-based nanostructures in plant disease diagnosis and control, as well as their safety in the agroecosystem, are discussed [17]. The development of antifungal nanohybrid agents containing conjugates of organic or inorganic compounds, biological components and biopolymers was researched in order to generate cheaper, more dependable and effective product(s) against most fungal infections of plants and animals [18,19]. Metal-bionancomposites such as Cu-Chit/NCS hydrogel are novel nano-fungicides created by metal vapor synthesis (MVS) that are used in food and feed to promote plant defense against toxigenic fungus, such as *Aspergillus flavus* linked with peanut meal and cotton seeds [20].

In conclusion, Fungal Nanotechnology 1 and 2 provides an updated and comprehensive knowledge dealing with the green and sustainable production of metal and organic-based nanostructures by various fungal species. In addition, intracellular and extracellular mechanisms will be investigated, focusing on fungal nanotechnology applications in biomedical, environmental and agri-food sectors. FN is still in its infant stage; therefore, significant studies should be focused on this area; plants, animals and humans will benefit greatly, and efficient and ecologically friendly approaches should be created.
